# Knowledge and reporting of adverse events following childhood immunization (AEFI) among health workers and caregivers at Mengo Hospital (2021), Kampala, Uganda: A mixed-methods study

**DOI:** 10.1371/journal.pgph.0004827

**Published:** 2025-07-08

**Authors:** Benjamin Watyaba, Lucy Chimoyi, Ivana Knezevic, Oliver Ombeva Malande, Hamza Kalutte, Henry Bazira, Faith Kewaza, Florence Adong, Oliver Nankonyoli, Bernard Kikaire

**Affiliations:** 1 Eastern Africa Consortium for Clinical Research, Uganda Virus Research Institute, Entebbe, Uganda; 2 University of Lausanne, Lausanne, Switzerland; 3 Implementation Research Division, The Aurum Institute, Parktown, Johannesburg, South Africa; 4 Administration Department, East Africa Centre for Vaccines and Immunization, Kampala, Uganda; 5 Social Science Department, Makerere University, Kampala Uganda; 6 Pediatric Department, Mengo Hospital, Kampala, Uganda; 7 Internal Medicine Department, Kirrudu Referral Hospital, Kampala, Uganda; PLOS: Public Library of Science, UNITED STATES OF AMERICA

## Abstract

Vaccines used in National Immunization Programmes (NIPs) are known to be safe and effective if used correctly, however, no vaccine is completely risk-free as adverse events occasionally occur after immunization. The World Health Organization (WHO) recommends adverse event following immunization (AEFI) surveillance for all vaccines as a critical tool to monitor safety and address potential concerns. However, the factors contributing to Uganda’s inability to meet global vaccine safety indicators remain unclear. This study assessed the level of knowledge and reporting of AEFI of children among healthcare workers and caregivers at Mengo Hospital, Kampala. A cross-sectional mixed-methods study involving three focus group discussions, each consisting of five caregivers, eight key informant interviews with healthcare workers (HCWs) and 388 face-to-face interviews with caregivers was carried out. Caregivers accompanying their children for their routine vaccination visits were invited to participate in the study. We descriptively summarized data using measures of central tendency, dispersion, and frequencies. Crude and adjusted measures of association between predictors and AEFI reporting were assessed using Pearson’s Chi-square test, and binary logistic regression analysis respectively. Thematic analysis was used to identify behavioral, personal, and social patterns influencing knowledge and reporting of adverse events following immunization among caregivers and HCWs. Approximately one-third of caregivers (130, 33.5%) reported that their children experienced an AEFI and reported it to a healthcare facility. Significant predictors of AEFI reporting included having a secondary education (aOR = 3.56, 95% CI: 1.12-11.30), tertiary education or higher (aOR = 8.52, 95% CI: 2.09-34.81), being older than 35 years (aOR= 18.77; 95% CI: 2.34-150.60) and unmarried (aOR = 3.27, 95% CI: 1.51-7.12). While most HCWs had good AEFI knowledge, they lacked awareness of the national reporting system. Strengthening training for HCWs and raising awareness among caregivers is essential to improve AEFI surveillance.

## Background

Immunization is a strategy for childhood survival and only second to clean water in terms of public health impact [1]. Vaccine use in addition to other factors has led to the eradication of diseases like smallpox [2], and wild poliovirus type 2 and 3 certified in 2015 and 2019 respectively. The under-5 mortality has reduced by 24% between 2010 and 2018, and it is projected that 122 million premature deaths will be prevented by vaccination globally over the lifetime of people born between 2000 and 2030 [3,4]. Although all vaccines used in National Immunization Programmes are known to be safe and effective, no vaccine is completely risk-free and adverse events occasionally occur after an immunization. Any unwanted occurrence to a vaccine recipient in form of a sign, symptom, disease, or abnormal laboratory finding which does not necessarily have a causal relationship is termed an adverse event following immunization (AEFI) [5]. Special interest is therefore required for vaccine pharmacovigilance to rapidly detect, report, and assess potential risks and provide targeted and tailored communication to the public. Activities that increase confidence in vaccines and maintain optimal coverage of the population at risk are needed [6–8].

AEFIs are rare, however, according to the WHO, all adverse events that are of concern to the caregiver should be reported regardless of severity. Alleged vaccine quality and safety issues must be dealt with rapidly and effectively [5,9,10]. Failure to do so can undermine confidence in a vaccine and ultimately have serious consequences for immunization coverage. For example, the delayed eradication of polio in certain countries was because of lack of trust in the vaccine and program [11]. The Global Vaccine Safety Initiative (GVSI) was established in 2011 with the primary objective of AEFI detection and ensuring that vaccine safety monitoring occurs even in low-resource settings. The Global Advisory Committee on Vaccine Safety (GACVS) proposed a reporting rate of at least 10 AEFIs per 100,000 surviving infants as the vaccine safety indicator for the immunization agenda 2030 [12]. The global average AEFI reporting ratio in 2015 was 549 reports per 100,000 surviving infants. Though the number of countries with AEFI reporting ratios greater than 10 increased from 8 (4%) in 2000–81 (42%) in 2015, this is still sub-optimal possibly given the wide variation in the reporting trends across regions [13]. Reporting of AEFI varies in the WHO regions; with 60% of American countries reporting at least 10 AEFI per 100,000 surviving infants, followed by 55% in the European Region, 43% in Eastern Mediterranean Region, 33% in Western Pacific region, 27% in South-East Asia region and 21% in African Region [13]. Studies from other countries have cited lack of training and fear of personal consequences, workload, and complicated reporting system as reasons for low knowledge and reporting respectively [14–16]. In spite of these, the reasons for failing to meet the vaccine safety indicator are not known in Uganda to the best of our knowledge.

In spite the remarkable achievements of the Uganda National Expanded Programme on Immunization (UNEPI), numerous challenges remain in identifying, reporting and management of AEFI by both caregivers and healthcare workers [17–19]. Fear of AEFI was cited as one of the barriers to the effective uptake of vaccines in Hoima district, western Uganda [17]. In 2019, the Ugandan government responded to a measles outbreak by rolling out the measles-rubella vaccine to over 19.1 million children were vaccinated. Despite this exercise, UNEPI registered only 99 cases of AEFI which was below the safety indicator and the rate of case-based serious AEFI reported during the campaign was 2.2 per 1000,000 doses [20]. Furthermore, Mengo hospital administer vaccination to at least 300 children monthly but data on AEFI is mostly unavailable as per the hospital records. It is unknown whether caregivers are unaware of the importance of reporting AEFIs, or healthcare workers are not documenting this information. To address this gap in one hospital in Kampala, Uganda, we conducted a mixed-methods cross-sectional study to determine the knowledge and reporting of AEFIs among healthcare workers and caregivers at Mengo hospital in Kampala, Uganda between August and October 2021.

## Methods

### Study design and setting

We conducted a cross-sectional sequential mixed methods study involving healthcare workers (HCWs) and caregivers of children accessing immunization services at Mengo hospital. Mengo hospital a private not for profit is one of the first hospitals to operate in Uganda and is a teaching hospital of Uganda Christian University Medical school located in Kampala Uganda. It provides free immunization services and more than twelve medical specialties with a bed capacity of 350. The immunization clinic is located at Mpereza complex and offers all the recommended childhood vaccines including HPV. The monthly number of babies vaccinated range from 300 for DPT3–485 for BCG.

### Study participants

We recruited respondents who were caregivers of children aged 0–24months of children born within the hospital and surrounding areas of Kampala and healthcare workers (HCWs) at the immunization clinic at Mengo hospital in the month of September to October 2021, had provided written informed consent and willing to be interviewed and audio-recorded. Caregivers were defined as either parents or any other guardian responsible for providing care to the child. We excluded caregivers attending their first immunization visit, as they may not have had any experience with procedures for reporting AEFI to HCWs. Additionally, HCWs who had worked at the clinic for less than six months were excluded, as we aimed to interview individuals with knowledge and experience in working within vaccination clinics.

### Sample size and sampling

Using the Kish Leslie formula with a hypothesized level of knowledge and reporting of AEFI at 50% for maximum sample size calculation, level of significance at 5%, desired level of power at 80% and 10% non-response rate, a sample of 430 participants was obtained for the quantitative component.

Consecutive sampling technique was used to select caregivers for the quantitative study while both HCWs and caregivers were purposely selected to participant in qualitative study. Twenty-three individuals were purposively selected for participation in qualitative sub-study. The qualitative sample size was based widely on accepted estimates from qualitative methodology. Data saturation will be assessed when the data can no longer produce new themes and it has been previously estimated that saturation may happen after 20 interviews [21].

### Data collection

Trained research assistants administered pretested standardized questionnaires to caregivers in waiting areas in the months of September and October 2021. We used questionnaires to collect data on sociodemographic, knowledge and reporting of AEFIs. Respondents were requested to provide the sources of knowledge information about AEFIs. The data collection tools were informed by World Health Organization (WHO) manual for surveillance of adverse events following immunization [22]. The tools were pre-tested on caregivers not involved in the main study and no subsequent modifications were made after. Questionnaires were administered by a research assistant in the caregivers’ preferred language at the vaccination waiting area. Translations to Luganda and back-translations ensured standardization. Qualitative data were collected through Focus Group Discussions (FGDs) conducted with caregivers and Key Informant Interviews (KIIs) with HCWs and inline immunization program managers.

**Focus groups discussions and key informant interviews:** all FGDs and KIIs were conducted in the waiting room in the afternoon when patients had left. The FGDs and KIIs were audio recorded and conducted in Luganda and English respectively. The interviews were conducted by a well-trained moderator (HB) with master’s level and four years of experience, alongside research assistant (FK) with Honors degree. None had a prior relationship with the study participants. All participants were provided with detailed study information before giving their written informed consent. Participants for FGDs were caregivers recruited through purposive sampling to achieve maximum variation in the sample. Three FGDs were conducted, each group consisting of five participants. The duration of FGDs were between 60–80 minutes, with saturation of data achieved for all themes identified. All the eight HCWs at the immunization clinic participated in KIIs. KIIs included nurses who had been working for at least six months in the immunization clinic and immunization program managers. Qualitative data was collected under two main themes: knowledge on AEFI and reporting of AEFI until saturation of themes was reached.

### Data analysis

The data entry screen was designed using Epi-data version 3.1 [23]. Entered data was cleaned with Microsoft Excel application and then exported into Stata 17 (StataCorp LLC) for analysis of quantitative data. Descriptive statistics (frequencies, percentages, medians with interquartile ranges (IQR) were used to summarize participant characteristics. Pearson’s Chi-square test/fisher’s test was used to determine associations between each predictor variable and dependent variable which was reporting of AEFI, and binary logistic regression analysis was used to establish relationships in multivariate data analysis. Adjusted Odds Ratios (aOR) and their 95% confidence intervals (CI) were reported. Significance was determined at 5%.

All interviews were transcribed verbatim and translated to English for audio recordings in local language by trained RAs (HB and FK). To analyze the qualitative data, we used thematic analysis and deductively developed codes based two themes: 1) knowledge of AEFIs and 2) Reporting of AEFIs. The codes were developed into subthemes.

### Ethical approvals

The study was carried out according to National guidelines and International Declaration of Helsinki (ICH-E6). The study protocol was approved by regulatory ethics review committee of Mengo hospital (MH/REC/44/06-2021), after review by University of Lausanne. We further received written consent from the respondents prior to their participation and audio-record during the interviews. Confidentiality and privacy were ensured by use of password protected database.

## Results

### Socio-demographic characteristics of caregivers of children attending an immunization clinic at Mengo Hospital Kampala, 2021

Of the 388 caregivers enrolled, majority were mothers of the children (n = 361, 93.0%), mean age was 28.74 years (Standard Deviation± 5.65). The youngest respondent was found to be 19 years. Most respondents were married (n = 278, 71.7%), had a secondary school education (n = 233, 60.0%), Anglican (n = 194, 50.0%), unemployed (n = 213, 54.9%) and had parity less than or equal to two children (n = 266, 68.6%). About half of the caregivers (n = 200; 51.6%) accompanied male children and most caregivers (n = 284, 73.2%) accompanied children below 14 weeks (Table 1).

**Table 1:** Socio-demographic characteristics and reporting of AEFI among Caregivers of children attending Immunization clinic at Mengo Hospital Kampala, 2021.

Characteristics	Has ever reported an AEFI		All (n = 388)	p-value
	No (n = 76)	Yes (n = 312)		
**Respondent**				
Parent	71 (19.7)	290 (80.3)	361 (93.0)	1.000
Caregiver	5 (18.5)	22 (81.5)	27 (7.0)	
**Respondent Age**				
<25 Years	29 (24.0)	92 (76.0)	121 (31.2)	0.001*
26–35 Years	46 (20.7)	176 (79.3)	222 (57.2)	
>35 Years	1 (2.2)	44 (97.9)	45 (11.6)	
**Marital status**				
Married	61 (22.0)	217 (78.1)	278 (71.7)	0.031*
Not married	10 (10.9)	82 (89.1)	92 (23.7)	
Previously married	5 (27.8)	13 (72.2)	18 (4.6)	
**Level of education**				
No education	6 (37.5)	10 (62.5)	16 (4.1)	0.012*
Primary	21 (27.6)	55 (72.5)	76 (19.6)	
Secondary	43 (18.5)	190 (81.6)	233 (60.1)	
Tertiary & above	6 (9.6)	57 (90.5)	63 (16.3)	
**Religious affiliation**				
Anglican	55 (19.8)	223 (80.3)	278 (71.7)	1.000
Non-Anglican	21 (19.1)	89 (80.9)	110 (28.4)	
**Employment status**				
Employed	29 (16.6)	146 (83.4)	175 (45.1)	0.199
Not employed	47 (22.1)	166 (77.9)	213 (54.9)	
**Parity**				
<=2	56 (21.1)	210 (79.0)	345 (88.9)	0.335
>2	20 (16.4)	102 (83.6)	43 (11.1)	

* p < 0.05 statistically significant

Abbreviation: n, number of participants.

Slightly above half of the respondents identified health education talks (n = 220, 56.6%) at the health facility as their main source of knowledge information concerning AEFIs and vaccination, followed by a family member (n = 192, 49.4%), radio/television (n = 156, 40.1%) and lastly friends (n = 148, 38.0%).

**Fig 1:**
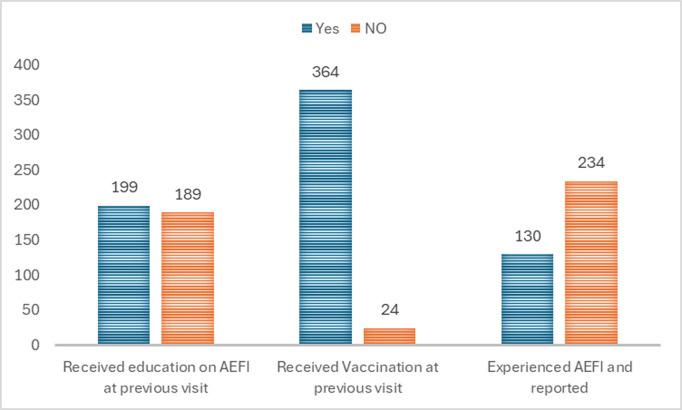
Summary of vaccination status, knowledge and reporting of AEFI among caregivers as per the last visit.

Among the 364 caregivers whose children received vaccinations at the previous visit, 130(35.7%) reported that their children experienced reactions after immunization and subsequently reported to the hospital (*See* Fig 1 *below*).

### Factors associated with reporting of AEFI

At multivariate analysis, the final model only included factors that were statistically significant (p-values < 0.05). At multivariate analysis, association were established between respondents with increased level of education: with secondary (aOR = 3.56, 95% CI: 1.12-11.30), and tertiary and above (aOR = 8.52, 95% CI: 2.09-34.81); respondent age of greater than 35years (aOR = 18.77, 95% CI: 2.09-150.60) and respondents who were not married (aOR = 3.27, 95% CI:1.51-7.12) (Table 2).

**Table 2:** Factors associated with reporting of AEFI by caregivers at Mengo Hospital Kampala, 2021.

Variables	cOR [95%CI]	p-value	aOR [95%CI]	p-value
**Level of education**				
No Education	1			
Primary	1.57 [0.51–4.86]	0.433	2.10 [0.62–7.11]	0.253
Secondary	2.65 [0.91–7.69]	0.073	3.56 [1.12–11.30]	0.031*
Tertiary & above	5.70 [1.53–21.25]	0.010*	8.52 [2.09–34.81]	0.003*
**Age**				
<=25	1		1	
26–35	0.57 [0.24–0.96]	0.155	0.70 [0.39–1.26]	0.234
>35	1.45 [1.84–26.85]	0.045*	18.77 [2.34–150.60]	0.006*
**Marital Status**				
Married	1		1	
Not Married	1.34 [1.28–11.54]	0.018*	3.27 [1.51–7.12]	0.003*
Previously Married	0.19 [0.04–1.11]	0.273	0.35 [0.09–1.24]	0.104

### Socio-demographic characteristics of participants in the qualitative sub-study

Of the 23 individuals who were interviewed, these included eight HCWs and 16 caregivers seeking immunization services at the immunization Clinic in Mengo Hospital.

Eight interviews were conducted with key informants who were nurses, line managers and focal persons in immunization. All participants had served for at least one year in immunization related services, majority had at least a diploma (87.5%; 7/8), 62.5% (5/8) had received some training in AEFI surveillance, 62.5% (5/8) were female and 87.5% (7/8) were of age ≥ 30 years. Three FGDs with a total of 15 caregivers were conducted. Most of the participants in the FGDs were not married (66.6%; 10/15), aged ≤30 years, and had at least secondary education.

**Themes on adverse events following immunization**: the findings from the FGDs and the KIIs are presented based on the two main themes that emerged from the data analyzed, with verbatim quotations from participants, presented in italics and enclosed in quotation marks. Each quotation is followed by a notation (FGD-P or KII-P) to identify the source of data, the participant and his/her sociodemographic characteristics.

#### Theme 1: Knowledge of AEFIs.

**a) Definition of AEFI:** From KIIs, most participants knew what an AEFI is. They described it as an abnormal occurrence that happens to a baby after immunization. They identified them as: an abscess at the site of injection, a fever, inflammation at the site of injection, occasional vomiting, body rash, allergic reactions and sometimes refusal to breast feed. Some study participants further categorized AEFIs as both minor and severe. Minor AEFIs were identified to be manageable at home by the caregiver if they occurred, whereas severe adverse events were reported to require professional management in a health facility.

“…*AEFIs can been categorized into both minor and severe. The minor events are the ones that can easily be managed, even at home. As health workers we normally only consider the severe events for example, convulsions*.” (KII 4 HCW, Mengo hospital).

Minor AEFIs were identified to be mild fevers, mild diarrhea, vomiting, regurgitation, pain, crying and redness at site of injection and severe AEFIs included stiffness of the baby, limited movement of limbs, sepsis, abscesses, swelling of legs, high fever, convulsions, limping, and failure to breast feed. However, it was pointed out that severe AEFIs rarely occurred.

**b) Management of AEFI:** According to participants in FGDs, management techniques of AEFIs varied depending on its severity in the child. Whereas majority agreed that minor AEFIs were easy to manage and could be managed at home by the care giver, severe AEFIs must be managed by a health worker in the health facility. They further explained that for example, a mild fever is managed by either applying a tepid sponge or giving the infant a warm bath, the swelling at the site of injection by applying a cold compress and exercising the limb in case of a stiff limb.

“…*we are usually advised by HCWs to exercise the limb in motions of cycling a bicycle while the infant rests on its back. This limb exercise enables the vaccine to move within the muscles and thereby restoring flexibility and free movement*” (Primary caregiver2, FGD-1).

Also, participants in all FGDs agreed that minor AEFIs such as vomiting majorly occurred when oral vaccines were administered. Therefore, to mitigate this, mothers/caregivers are encouraged not to feed the infant ten minutes before and after vaccination such that the vaccine can be absorbed into the body. In case the baby started vomiting after vaccination, the infant is laid on its side to avoid choking on the vomit. Caregivers are also encouraged to breastfeed the baby in case of experiencing pain and crying too much.

According to health workers, caregivers are discouraged from giving any form of medication to infants before and after vaccination. This is because some medicines may interfere with effectiveness of vaccines. However, if symptoms persist then they advise the caregivers on home management before going to hospital.

*“…we give Paracetamol syrup, and Ibuprofen to relieve pain and fevers; antibiotics such as amoxicillin syrup for sepsis and abscesses, and Zinc to manage diarrhea and vomiting. If AEFIs persist, caregivers are advised to take the infant to the nearest health facility.”* (KII 5 HCW, Mengo hospital)

**C) Coincidental illnesses falsely attributed to vaccines:** Majority of participants in KIIs were aware of coincidental illnesses which may pass for AEFIs. They defined them as those underlying infections that might not be detected before immunization but present after immunization with no correlation with effects of the vaccines. These illnesses are normally falsely attributed to the vaccines because of the temporal relationship. Illnesses mentioned include malaria, bacterial and viral infections. Some caregivers revealed that some of these complications are caused by caregivers mishandling immunization sites in infants.

“… *once a baby is immunized, we as caregivers tend to go against instructions and apply local remedies for example, massaging the site of injection with herbs which leads to complications. And when asked by health workers we deny and blame them or the vaccine*.” (Primary caregiver 1, FGD-2).

**D) Source of knowledge on AEFIs:** HCWs were asked whether they have received any training in management of AEFIs, some reported to have attended a one-day seminar on AEFI surveillance. However, all reported getting some knowledge on AEFIs while in training institutions. On the other hand, caregivers reported to have received this knowledge from health education talks conducted by health workers during ANC and before immunization followed by friends and peers within the communities.

“…*nurses teach us before they vaccinate, the problem most caregivers come late when the teachings have ended.”* (Primary caregiver 4, FGD-3).

#### Theme 2: Reporting of AEFIs.

**a) General knowledge on reporting of AEFIs:** Most KII participants agreed that reporting means monitoring, tracking, and gathering events about immunization and sharing these facts with the concerned persons. Whereas most didn’t know of the existence of a reporting system specifically for AEFIs, they acknowledged an existing form from National Drug Authority (NDA) that collects information for both adverse drug reaction (ADR) and AEFI which many had never seen or used before. The researcher probed into the reason this tool isn’t being used and if they were familiar with national AEFI reporting system. Almost all participants agreed that whereas minor AEFIs are managed by the caregivers at home, and severe AEFIs rarely occurred at immunization clinic, they are not aware of the national AEFI reporting system. Similarly, there are no standard operating procedures (SOPs) regarding reporting of AEFIs.

“…*we have a book for reporting events in the office although we have never been utilized it. We are not aware of the offices to send the forms after filling*.” (KII 7 HCW, Mengo hospital).

In addition, the KII respondents were asked whether they had ever reported an AEFI. Majority of the respondents mentioned that they had never reported AEFI to authority because they don’t know how to fill in the ADR forms. Secondly, majority of health workers said that events rarely occurred at the immunization clinic and that they occasionally receive complaints from care givers during return visits for immunization when the event has resolved hence no need to report.

“…*most parents tell us about AEFIs when they come back for their next visit. So, we don’t bother to report since they could have resolved*.” (KII 1 HCW, Mengo hospital).

**b) Factors affecting reporting of AEFI:** Majority of participants agreed that there is need to identify the issues hindering reporting of AEFIs in health facilities. Among those mentioned were availability of reporting platform, on-station mentors for capacity building regarding immunization services, frequency of occurrence of AEFIs, availability of facilitation for follow-up after vaccination, lack of access to further training opportunities by staff, inadequate support supervision, and lastly inadequate follow-up by implementation Partners (IPs) like UNEPI, Ministry of Health (MoH) and Kampala Capital City Authority (KCCA)

“…*we have a form that is rarely utilized. These people monitoring the AEFIs only get serious when there is an on-going campaign on immunization. The UNEPI people are supposed to streamline this process, but I think they are not taking it very seriously. Therefore, they should come and conduct trainings, provide all the necessary equipment and ensure that the reporting materials are used*.” (KII 8 Focal person, KCCA).

c) **Measures to improve reporting of AEFIs:** The most cited measures to improve AEFI reporting were the need to enhance capacity building for proper reporting AEFI. Other measures include improving on-site mentoring & support supervision, providing vaccinators and other health workers with more training in vaccination, provision of AEFI reporting materials, routine monitoring and follow-up by AEFI surveillance teams and provision of facilitation for health workers to conduct follow-ups on clients since majority of these AEFI happen at home. Furthermore, participants agreed that digital reports would greatly improve reporting of AEFIs in the hospital as it is easier to fill and utilize, and they can be accessed from anywhere making real-time reporting easy.

“…*we have no problem to report AEFIs. We need to be trained on the available platforms.”* (KII 2 HCW, Mengo hospital)

“… *The issue with a paper-based system is, once the forms are filled out, where and how do they submit them?”* (KII 5 immunization support person, KCCA)

## Discussion

This study found that knowledge levels and reporting of AEFI among caregivers was sub-optimal. Though majority of the healthcare workers could define and identify AEFI, most were not aware of the reporting process.

Our findings are consistent with other studies globally documenting poor level of knowledge and reporting of AEFIs [24,25]. For example, Mutata et al noted 39% of the caregivers had children who had experienced an AEFI and 45% of the health workers had encountered AEFIs but none had been notified [25]. These findings have implications on the national surveillance of AEFIs and warrants more effort improve reporting. For example, health education at immunization clinics and family members were noted as the most common sources of AEFI knowledge. Thus, it is necessary to develop training and educational programs in order to increase awareness of caregivers and HCWs involved in child health toward reporting of AEFIs [26].

The study also found that caregivers with a secondary education or higher, as well as those aged 35 years and above, were more likely to report AEFIs. This suggests that educated and older caregivers may be more attentive to changes in their children following immunization and are more likely to anticipate potential reactions from vaccinations and thus report.

The low reporting of AEFI noted in this study is a perilous trend in capturing useful information about vaccine safety. The Ministry of Health has a paper-based tool for the identification and reporting of AEFIs. However, the study established that HCWs were not using them for their intended purpose because they were not aware of the procedure for reporting. Lack of capacity building for proper reporting, on-site mentoring & support supervision and complex reporting system were cited as some of the reasons by healthcare workers to report and this was also noted by Raymond Aborigo et al and Omeleke SA et al [26,27]. Most caregivers were reluctant to report mild AEFI that are managed routinely at home. This was also noted in another study in Zimbabwe by Mutata et al. [25].Thus, capacity building through trainings for both caregivers and healthcare workers are still needed [14]. Furthermore, participants agreed that electronic reporting would improve the shortcomings of paper-based system.

### Study strengths and limitations

This study conducted in Uganda’s Capital city, offers a comprehensive perspective on AEFI knowledge and reporting among frontline HCWs, health managers, and caregivers. The study’s strength lies in the triangulation of quantitative and qualitative findings, enhancing the reliability of the interpretation of findings. Our study is not without limitations and the findings should be interpreted with caution. For example, the study involved asking about untoward events experienced at 0,6,10, and 14 weeks, thus there was a likelihood of recall bias from both caregivers and HCWs. Besides, the self-reported nature of the data may have led to reporting and selection bias however this was reduced by random sampling of participants. Finally, since this was a cross-sectional study, we were unable to make any causal relationship between AEFI knowledge, presence of AEFI and likelihood of reporting, as the study design only captures associations at a single point in time and lack of generalizability since data was collected within Kampala and surrounding areas. Despite these limitations, the study provides valuable insights into AEFI knowledge and reporting practices.

## Conclusions and public health implications

AEFI reporting among caregivers was suboptimal, and level of education and age were the key factors influencing reporting behavior. While most HCWs had good knowledge of AEFI, they lacked awareness of the national reporting system highlighting a critical gap. To enhance the effectiveness of AEFI surveillance, efforts should be focused on increasing awareness and training HCWs on proper reporting procedures, alongside targeted interventions to encourage AEFI reporting among caregivers with lower education levels or other at-risk groups.
